# Respiratory and physical health consequences in older adults in a high-risk volcanic area: Comparison of two rural villages

**DOI:** 10.1371/journal.pone.0310659

**Published:** 2024-09-19

**Authors:** Vicente Benavides-Cordoba, Andres Suarez, Diana Guerrero-Jaramillo, Melissa Silva-Medina, Jhonatan Betancourt-Peña, Mauricio Palacios-Gómez

**Affiliations:** 1 Facultad de Salud, Universidad del Valle, Cali, Colombia; 2 Instituto Departamental de Salud, Pasto, Colombia; 3 Fundación Universitaria Maria Cano, Cali, Colombia; 4 Institución Universitaria Escuela Nacional del Deporte, Cali, Colombia; Universidad San Francisco de Quito, ECUADOR

## Abstract

**Introduction:**

Volcanism is an important natural producer of pollution that impacts health and the quality of the environment. Lung changes caused by exposure to volcanoes have been previously studied. However, limited information exists regarding the effects of prolonged exposure to volcanic compounds. So, this study aimed to analyze the pulmonary effects and stress tolerance in older adults for chronic exposure to the volcanic ashes of the Galeras volcano.

**Methods:**

A descriptive cross-sectional study of association included rural inhabitants aged over 60 years from Genoy, a village located in a high volcanic hazard zone of Galeras volcano, 2603 meters above sea level. Those in this group, called exposed, were contrasted with a sample of El Encano inhabitants with similar socioeconomic and cultural characteristics. Both villages belong to the rural area of San Juan de Pasto in Colombia.

**Results:**

It was found that of 31 exposed participants, 18 had obstructive alteration, and in the control group, it was found that of 31 subjects, 6 presented this alteration. The difference between the two groups was significant (p<0.001). A similar situation occurred with distal airway obstruction assessed with the forced expiratory flow of 25–75%. No significant differences were found in restrictive alteration between the exposed and unexposed groups.

**Conclusion:**

Chronic exposure to volcanic compounds has generated obstructive changes in the population, and these changes were greater in number and severity than those in the control group of unexposed people.

## Introduction

Historically, communities have been exposed to disasters such as volcanic eruptions and earthquakes. In 2023, 86,473 people died due to natural hazards as a result of 399 reported disasters [1]. Volcanism is an important natural producer of pollution that impacts health and the quality of the environment [2]. Pollution measurements of active volcano environments exceed expectations; volcanoes are responsible for expelling of 0.13 to 0.44 billion metric tons (gigatons) of carbon dioxide per year [3]

The association between volcanoes and respiratory diseases has been studied for over 40 years. In 1980, the explosion of Mount St. Helen, an andesitic volcano, became a concern for pulmonologists, who feared that the SiO_2_ found in the ’ ’volcano’s ash could cause acute silicosis or pneumoconiosis. Particle size that was as small as 1 micron was of concern, particles smaller than 5 microns were known to have the potential to cause harm in occupational settings. Acute manifestations were seen in patients with Chronic Obstructive Pulmonary Disease (COPD) and asthma but were reported to be in the same amount expected for smog, with minor irritation as the first cause of emergency room visits; nine deaths were reported with autopsy findings suggesting that the inhaled ash was still hot. The ashes were later described as having 70% silica crystals but not in a toxic form. That experience concluded that ash inhalation was probably not a “major respiratory problem” [4, 5]. Acute exposure to volcanic ash and gases has been studied during major explosive events. Understanding eruption patterns of volcanoes is necessary for assessing exposure risks. Volcanoes are classified as andesitic, basaltic, and rhyolitic. Basaltic volcanoes primarily produce lava with little ash, causing less damage and being more predictable. Rhyolitic volcanoes are highly destructive but erupt rarely [6]. Andesitic volcanoes, with 57–63% silica by weight, are unpredictable and can have prolonged ash events [7]. These volcanoes cause chronic exposure to gases and particulates, not only during major eruptions, and release harmful radon [8]. The Galeras volcano (Smithsonian Institution’s Global Volcanism Program, volcano number 351080) is an andesitic volcano [9].

Lung changes caused by exposure to volcanoes have been previously studied in schoolchildren [10], in cellular models, as well as *in vitro* models that evaluate bacterial growth [11]. In other populations, specifically in older adults, it has been identified that they are particularly susceptible to this exposure [12]. In the case of the 2002 Mount Etna eruption, a temporary increase in cardiovascular morbidity was identified, which is said to have been possibly secondary to acute stress [13]. During the 2014–15 Holuhraun eruption in Iceland, a significant fissure eruption, it was observed that older adults were the demographic most inclined to seek care from primary healthcare services following the event [14].

Physiological and metabolic changes associated with aging suggest that exposure to volcanic ash, may have similar effects to environmental pollution, which has been identified as one of the 12 key risk factors in old age. Such exposure can lead to respiratory and cardiovascular problems and may even increase the risk of dementia [15]. However, there is scarce information regarding the effects of chronic exposure to volcanic compounds. So this study aimed to analyze the pulmonary effects and tolerance to the effort in older adults for chronic exposure to the volcanic ashes of Galeras volcano in two rural villages. These results will enable the relevant authorities to strengthen the basis for decision-making to prevent immediate damage caused by acute exposure and implement measures that minimize potential respiratory sequelae resulting from chronic exposure.

## Materials and methods

**Fig 1:**
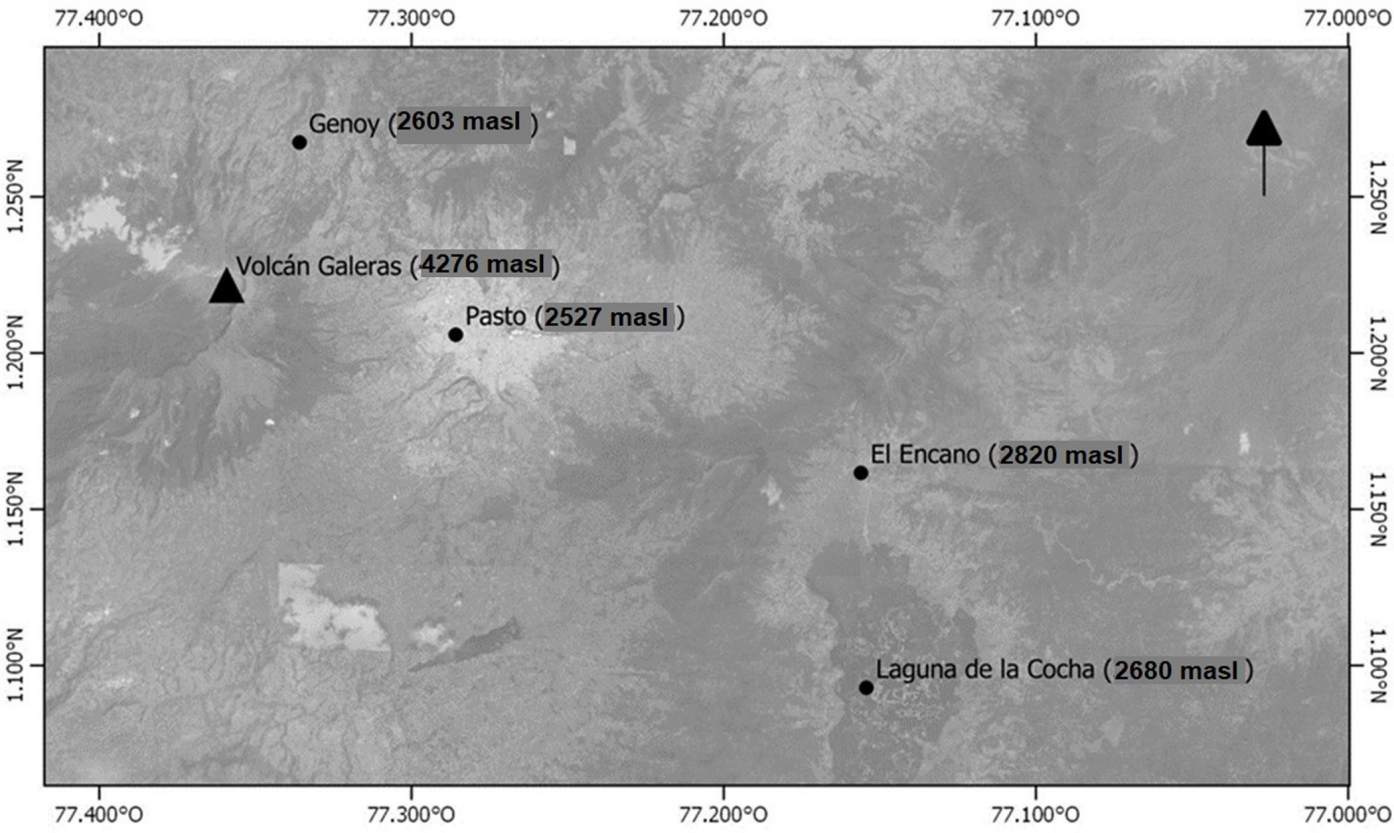
Geographical location.

A cross-sectional study of association included inhabitants aged over 60 years from Genoy, a village located in a high volcanic hazard zone of Galeras volcano, at 2603 meters above sea level; those in this group, called exposed, were contrasted with a sample of inhabitants from El Encano, with similar socioeconomic and cultural characteristics. El Encano is a village located at 2,803 meters above sea level, close to Laguna de la Cocha, a body of water with an area of 40 km^2^ that serves as a sink for the pyroclastic materials thrown by the volcano, but that is not located in the high volcanic hazard zone. Both communities belong to the rural area of San Juan de Pasto (Fig 1).

The Volcán Galeras (4276 meters above sea level) is located in the Nariño Department. Its coordinates are 1°13.31’ N and 77°21.68’ W, within the geographical domain of the Cauca-Patía inter-Andean depression. According to information from the Colombian Geological Service, the high-risk zone affected by volcanic gases corresponds to the upper parts of the volcano and the river channels originating from it or the slopes of the Galeras volcanic complex. These include the valleys of the Azufral, Chacaguaico, and Barranco rivers, and the streams Pailón, La Chorrera, Huilque, Maragato, Chorrillo, Agua Agria, El Vergel, Los Saltos, San Francisco, Mijitayo, Midoro, and Genoy, which is the village included in this study. masl: meters above sea level

These villages share common characteristics related to access to public services, sanitation, and public transportation. Their primary economic activity is agriculture, and they maintain a rural lifestyle, sharing cultural customs, including the Carnival of Blacks and Whites and ethnic identity, with a population primarily composed of indigenous and mestizo individuals. The climate in both areas is considered subtropical mountainous, with temperatures ranging from 10 to 18 degrees Celsius (°C).

People living in this area for at least ten years were admitted to the study; these participants had no history of chronic lung disease, diagnosed by spirometry. Participants were not excluded due to exposure to biomass or smokers.

Between January to December 2019, recruitment was carried out following international bioethics standards, with the ethical guidelines for health-related research with human beings CIOMS version 2017 and the resolution 8430 of 1993 of the Colombian Government that sets the regulations for research in this country. The research ethics committee of the municipal health agency endorsed the project.

To enroll the population, with the due permission of the health entities that attend these communities, the people attending the health centers of Genoy and El Encano were included. Informed consent was obtained in writing by the principal investigator before entering the investigation. The investigator answered all the participants’ questions, and the signed consent documents were stored in the University facilities. The principles of totality/integrity, respect for the person, non-maleficence and autonomy, beneficence, and justice/equity were considered.

### Instruments and materials

The following variables were collected: sociodemographic ones such as sex, age, place of origin, socioeconomic status and marital status; clinical variables such as exposure to volcanic compounds, exposure to wood smoke, smoking, visits to the emergency room in the last year, home oxygen, associated diseases, pharmacological treatment, level of physical activity, and body mass index (BMI) [16], associated symptoms such as dyspnea in activities of daily living according to the Medical Research Council (MMRC), chronic cough, pulmonary secretions, aerobic functional capacity (1 minute Sit to Stand Test), and pulmonary function (Spirometry FVC: Forced Vital Capacity, FEV_1_: Forced expiratory volume in 1 second and FEV_1_/FVC).

### 1-minute sit-to-stand test

The test was developed according to previous studies that established the protocols to be executed [17]. It was carried out with a standard chair of 46 centimeters; the individuals were instructed to accommodate their upper limbs crossed over the thorax and to get up and sit down from the chair as fast as possible during one minute. The number of repetitions, heart rate, respiratory rate, mean arterial pressure, oxygen saturation, dyspnea, and fatigue were recorded using the modified Borg scale [18]. The patients rested for at least 30 minutes before performing the test [19].

### Spirometry

The measurement was performed according to the American Thoracic Society (ATS) and the European Respiratory Society (ERS) guidelines with the Spirobank MIR II® equipment. In addition, the lower limits of LLN normality for FVC, FEV_1_, FEV_1_/FVC and FEF (Forced expiratory flow) 25–75% were taken as a reference to determine the incidence of obstructive alteration and restrictive alteration [20].

### Statistical analysis

The normality test with the D’Agostino Pearson test was applied to the variables of interest, those related to aerobic functional capacity and lung function. In the presence of a Gaussian distribution, the comparison of the results between the inhabitants of Genoy and El Encano through the Student’s t-test. In the absence of the normality assumption, the Wilcoxon test was used. A statistical significance level of 5% was considered.

Chi-squared test and Odds Ratio (OR) with a 95% Confidence interval (CI 95%) was made to identify the association between exposure and obstructive or restrictive alteration. The analysis of the data and the graphs were carried out with the GraphPad Prism Software, version 7.0.

## Results

A total of 62 participants entered the study, 31 in the exposure zone and 31 in the non-exposed site. Twelve participants in whom spirometry could not be performed were excluded due to poor understanding of test execution. Participants over the age of 60 were included in the study. A higher number of women were enrolled, with the average age being similar across study groups. The largest proportion of participants were exposed to biomass. This exposure is derived from the combustion of firewood used for food preparation; cigarette exposure was less than 25% in both groups. Regarding exacerbations, it was found that in the exposed group, there were 9 participants with respiratory exacerbations, compared to no exacerbations in the non-exposed group, being this the only baseline variable with a significant difference (p < 0.05). (Table 1). In clinical variables, it was obtained that the exposed group had an average of 19.03 +/- 0.62 repetitions, and the case group 19.71 +/- 0.98 repetitions in the result of the functional capacity measured with the sit-to-stand test, without statistical difference between the groups. The variables in which there was a difference were fatigue and exercise-related dyspnea, which were measured with the Borg Scale, which was determined/measured at the end of the test. Differences were also identified in lung function assessed with the FEV_1_/FVC ratio and the FEV_1_ (Table 2).

**Table 1:** Characteristics of the population.

VARIABLE	EXPOSED (n = 31)	NON EXPOSED (n = 31)
**Sex female: n (%)**	19 (61.3)	15 (48.4)
**Age: years (CI 95)**	70.29 (67.11–73.47)	73.68 (69.7–77.5)
**Weight: Kg (SD)**	66.6 (8)	66.9 (22.16)
**Height: M (SD)**	1.54 (0.09)	1.59 (0.11)
**BMI: (SD)**	27.8 (2.75)	26.66 (4.42)
**Respiratory**		
**Biomass exposure: n (%)**	28 (90.3)	27 (87.1)
**Cigarette exposure: n (%)**	7 (22.6)	7 (22.6)
**Respiratory exacerbations: n (%)**	9 (29)	0 (0)
**Chronic Cough: n (%)**	11 (35.5)	13 (41.9)
**Lung Secretions: n (%)**	7 (22.6)	4 (12.9)
**Dyspnea: n (%)**	11 (35.5)	13 (41.9)
**Time living in the zone: years (CI 95)**	50.16 (42–58.3)	60.65 (52.5–68.7)

* p < 0.05, CI: confidence interval. BMI: Body mass index, SD: Standard Deviation

**Table 2:** Clinical variables.

	Exposed n = 31	Non Exposed n = 31
**mMRC**	1.17 (0.15)	0.74 (0.14)
**Borg Fatigue**	1.67 (0.24)	1.03 (0.11)
Borg Dyspnea*	1.75 (0.23)	1 (0.26)
**Heart Rate**	81.3 (2.08)	83.68 (2.54)
**Mean arterial pressure**	92.37 (1.9)	92.27 (2.26)
**Oxygen saturation**	90.01 (1.93)	90.77 (0.61)
**Sit To Stand (Rep)**	19.03 (0.62)	19.71 (0.98)
**FVC (%)**	91.29 (4.22)	88.71 (3.58)
**FEV**_**1**_ (%)*	79.34 (5.45)	86.89 (4.51)
**FEV**_**1**_ /FVC*	64.69 (4.14)	77.13 (2.95)

* p < 0.05, mMRC: modified Medical Research Council, FVC: Forced Vital Capacity, FEV_1_: Forced Expiratory Pressure in 1 second.

The association between exposure and chronic lung disease is presented in Table 3. With respect to obstructive lung alteration, it was found that 18 out of 31 exposed participants had this condition. In contrast, only 6 out of 31 participants in the control group exhibited the same alteration. The difference between the two groups was statistically significant, with an OR of 5.76 and a CI 95% of 1.84 to 18.07 (p<0.001). A similar situation occurred with distal airway obstruction assessed with FEF 25–75%, with a predicted percentage of less than 60%; 13 people from the exposed inhabitants had this alteration compared to 4 people from the control group. No significant differences were found in restrictive alteration between the exposed and unexposed groups, with 3 and 5 cases with LLN of the FVC, respectively.

**Table 3:** Association between exposure and chronic lung disease.

	Exposed n = 31	Non-exposed n = 31	OR	CI 95%
**Obstructive changes**	18	6	5.76	1.84–18.07
**Restrictive changes**	3	5	0.55	0.12–2.56
**DAO**	13	4	4.87	1.36–17.36

OR: Odds Ratio, CI: Confidence interval, DAO: Distal airway obstruction

## Discussion

The Galeras volcano is an andesitic stratovolcano; its main crater is located at 4,276 m above sea level. It is considered an active volcano with main episodes of weak fumarole activity, ash emissions, and small-scale eruptions. Eruption periods have been recorded since 1988 [21].

The main objective in attempting these measurements is identifying hazardous events/situations in potentially active volcanoes [22]. Reactivation of the Galeras volcano began in 1988, with SO_2_ records of up to 5495 tons/day; six volcanic eruptions were recorded from 1992 to 1993. After that, there was a period of relative rest, with seismic events until 2004, when several small explosive eruptions were recorded, associated with the emission of ash and an increase in the emission of SO_2_ [21]. After this event, measurement techniques gradually changed due to the destruction of some measurement devices during the eruption. The fumarolic gases of Galeras volcano are composed of H_2_O, CO_2_, S (as SO_2_ and H_2_S) and HCl, and inert gases such as N, Ar, and He. Its exact composition was determined in 1997 by mass spectrometry. However, the Galeras volcano complex has several craters with different temperatures, and the proportions of gases vary between them. Total S and CO_2_ are the main components, with highly variable HCl and low concentrations of HF, H_2_O, and inert gases [23].

### Exposure criteria and health impacts

Few studies are available related to exposure to volcanic eruption products that consider the relative frequency of these events (volcanic eruptions). As an inclusion criterion, only persons with a minimum of 10 years of exposure were enrolled; this decision was made because there are no conclusive studies of what is the minimum time to define chronic exposure to volcanoes, especially considering that it is not a continuous exposure, because it is subject to volcanic activity. Furthermore, this exposure parameter is accentuated by aging, which accelerates lung injury and damages the cells responsible for maintaining pulmonary homeostasis [24]

Estimating the chronic effect of exposure to air pollution from volcanoes is difficult because, as in other studies on respiratory exposure, it takes decades to consolidate a chronic lung disease. For this reason, we decided to consider a minimum time of 10 years, a volcano with sustained activity over the last 20 years, and a population with accumulated lung exposure to other pollutants such as biomass and cigarettes and who live at high altitudes. With these characteristics, we would expect an increase in the population with COPD or interstitial lung disease without affecting functional aerobic capacity tests.

Exposure to particulate material from the volcano increased the number of cases of obstructive disease with compromised lung function. We were interested in the cases of restrictive patterns related to the quantities of substances expelled by the volcano, such as sulfuric acid, sulfur dioxide and hydrogen sulfide [23]. The data we found do not allow us to draw conclusions about the effect.

Exacerbations were significant compared to controls, and we believe that this indicator could have greater relevance in this type of study because it predicts the risk of death, and hospitalization and affects the patient’s therapeutic classification on the scale of the *Global initiative for the diagnosis*, *management*, *and prevention of chronic obstructive lung disease GOLD* [25].

We did not expect to find marked differences in functional aerobic capacity tests because this indicator is affected in patients with the diagnosed disease and advanced stages. There could be some controversy regarding the use of the sit-stand test and the 6-minute walk test, but studies on COPD and Intersticial Lung Disease (ILD) are equally reliable regarding oxygen consumption [26, 27].

This study is an analysis of the effects, as well as on health, symptoms and lung function associated with exposure to a volcanic environment in the air in a population that lives in an area adjacent to a volcano in a village of Nariño, Colombia, compared to the people of another town of the same city which does not present this type of risk. ’Considering that the people exposed to volcanic gases reside within a 35 km radius of the volcano, it is considered a high-risk area, which is corroborated by different studies evaluating the population in threat areas within similar radii of volcanoes [28]. This suggests that the results of this study are more related to exposure to the ambient air of the volcano over other harmful factors such as smoking and exposure to biomass.

The implications and importance of this study lie in the fact that about 10% of the world’s population resides in areas near active volcanoes that emit gases that are harmful to health, such as SO_2_, CO_2_, radon, H_2_S and other particles derived from acidic substances into the ambient air that favor air pollution, being a risk factor for respiratory symptoms and alterations in populations exposed to them [10, 29]. It is necessary to consider that the Galeras volcano is an active volcano that emits these same gases during its greatest activity. These populations are permanently exposed since their demographic transition to other municipalities is scarce. On the contrary, population growth is causing human settlements to increase more and more in the areas surrounding the volcano, which increases the risk for these people [30]. It should be clarified that adults aged over 60 years were admitted since they were the people who mostly met the inclusion criteria of exposure time, and considering that lung function shows deterioration concomitant with aging [31]. The deficiencies in cellular repair mechanisms are a distinctive feature of pulmonary aging. Inhaled exposures diminish the lung’s regenerative potential by inducing oxidative stress, DNA damage, epigenetic instability, telomere shortening, mitochondrial injury, and abnormal protein imbalance in key progenitor and structural cells. Accumulated damage in mesenchymal stem cells leads to apoptosis and stem cell depletion, while repeated insults in type II alveolar epithelial cells and lung fibroblasts contribute to cellular aging. This ultimately means that two factors come into play: aging and exposure.

In previous studies, researchers have described that the concentrations of SO_2_ in the risk zones of gas emanation from other volcanoes, measured during 24 hours, are close to 300–700 tons per day, and the concentrations in the most polluted areas are close to 75 ppb in the periods of maximum activity, down to 13 ppb in those of less activity; and even 10.1 ppb in some regions of the USA [32]. This situation is similar to that of the Galeras volcano, where the SO_2_ gas emission values in the last eruption period, measured in 2009, were close to 733 tons [21], which represents a similar exposure to that from other studies and, therefore, could cause similar conditions in the health status of populations residing in risk areas. This is supported since, according to Trisnawati I et al. [33], they state that short-term exposure induces asthma and bronchitis attacks, increasing cough, dyspnea, chest tightness, and wheezing, mainly due to irritation of the lining of the airways by finer particles, also, a worsening of other respiratory diseases such as chronic bronchitis. Those above may be related to the fact that over 75% of the participants cooked with biomass, which could increase the obstructive and distal airway deterioration observed in the spirometry. Finally, much longer exposure to volcanic emanations can cause retention of inorganic particles that are inhaled and generate fibrosis in the pulmonary interstitium; consequently, the chronic condition evolves with decreased distensibility, increased elastic recoil, and deterioration of the lung tissue. As a result, there is a greater cough due to the particles trapped in the airway, which forces coughing more frequently as a defense mechanism [33].

Studies have evaluated the effects on the health status of exposure to volcanic emanations; the main ones are related to short-term respiratory system disorders [34, 35], such as chest tightness, cough and eye irritation; these signs or symptoms were the most frequent in those exposed, compared to the ones who were not; in turn, these studies show that the symptoms get reduced over time. In contrast, this study evaluates the exposed population over more than ten years. It found the presence of symptoms, emergency visits and deterioration of lung function in the evaluated population, which is consistent with what was exposed in other long-term follow-up studies [36], in which it is indicated that these symptoms can persist up to 4 years after exposure; this can be explained by the fact that there is frequent exposure in the region to harmful particles, which contaminate the breathable air and cause deleterious effects on health. Other reported symptoms, such as eye and skin irritation, are unusual in the long term [36, 37].

### Preventive measures, relocation challenges, and public policy implications

The best measure to prevent exposure to particulate matter from volcanoes is to relocate the population from high-risk areas. But, in practice, there’s much resistance to this measure from the inhabitants and little will from the Government to implement it. For this reason, it is necessary to assess or estimate the effects of chronic exposure to volcano contamination. To achieve this, it is crucial to conduct an evaluation that considers urbanization in areas near volcanoes. The application of indices such as the Volcano Population Index, the Population Exposure Index, and the Volcanic Risk Coefficient are quantitative measures that can serve as important substrates for governments to make evidence-based decisions [38]. Although they do not guarantee residents’ acceptance, they strengthen negotiation arguments.

Furthermore, it is necessary to consider that several items must be met to favor a successful relocation: access to livelihood activities, the continuance of social networks, culturally appropriate housing that meets basic needs, and community participation throughout the design and implementation of the project. Decision-making authorities should consider this, as it could improve relocation policies for populations in areas of high volcanic threat.

Volcanoes’ impact on populations must be evaluated through public policies that include strengthening observatories through best practices. The guidelines have established these practices, which include designing networks to address volcanic threats, creating protocols and checklists, the capacity to collect data through alarms and models, organizing people through education and training, collaboration between government agencies, effective communication from public service offices, dissemination of measures, the use of social media, and finally, optimizing post-response measures by evaluating the results obtained.

One of the study’s limitations is its sample size, which restricts the generalization of results to larger populations. In addition to this, no children or population were linked to other comorbidities. However, it’s crucial to acknowledge that the participants had a specific age range and, notably, a minimum exposure of 10 years, providing insights into this particular population’s circumstances. Additionally, while participant-supplied information may be subject to bias, particularly regarding symptom reporting, it’s essential to recognize the objectivity and reliability of the administered tests (spirometry and sit-to-stand test). These tests contribute valuable, concrete data to the study’s findings, enhancing the overall credibility of the collected information.

Similarly, confounding factors such as occupational exposure or respiratory pathologies could be associated with genetic disorders or susceptibility. However, these factors could be minimized by considering that both groups shared similar economic activities, with agriculture being the main source of income in both communities. This balanced the studied groups.

## Conclusion

Chronic exposure to volcanic compounds has generated obstructive changes in the population; these changes were greater in number and severity when compared to the control group of unexposed people. Local health authorities should guide the entire population to develop protection strategies against gases and particulate matter, thereby preventing respiratory complications. Moreover, they should advocate for strategies to reallocate economic resources and ensure socio-environmental sustainability, facilitating the medium- and long-term relocation of residents who are hesitant to leave their homes. Besides institutional action, it is imperative to engage the population in decision-making processes to mitigate the acute consequences of natural hazards and proactively address the long-term effects of cumulative exposure.
